# 1-*n*-Decyl-5-nitro-1*H*-benzimidazol-2(3*H*)-one

**DOI:** 10.1107/S1600536811004399

**Published:** 2011-02-12

**Authors:** Younes Ouzidan, Y. Kandri Rodi, Sonia Ladeira, El Mokhtar Essassi, Seik Weng Ng

**Affiliations:** a Laboratoire de Chimie Organique Appliquée, Faculté des Sciences et Techniques, Université Sidi Mohamed Ben Abdallah, Fés, Morocco; b Service Commun Rayons-X FR2599, Université Paul Sabatier Bâtiment 2R1, 118 route de Narbonne, Toulouse, France; c Laboratoire de Chimie Organique Hétérocyclique, Pôle de Compétences Pharmacochimie, Université Mohammed V-Agdal, BP 1014 Avenue Ibn Batout, Rabat, Morocco; d Department of Chemistry, University of Malaya, 50603 Kuala Lumpur, Malaysia

## Abstract

The benzimidazolone part of the title mol­ecule, C_17_H_25_N_3_O_3_, is almost planar (r.m.s. deviation = 0.016 Å) and its mean plane is aligned at 7.9 (4) ° with respect to the mean plane of the nitro substituent. In the crystal, two mol­ecules are disposed about a center of inversion, generating a N—H⋯O hydrogen-bonded cyclic dimer with a *R*_2_^2^(8) graph-set motif.

## Related literature

For the crystal structure of 1-isopropenyl-1*H*-benzimidazol-2(3*H*)-one, see: Saber *et al.* (2010 ▶) and for 5-nitro-1-*n*-octyl-1*H*-benzimidazol-2(3*H*)-one, see: Ouzidan *et al.* (2011 ▶). For graph-set notation, see: Etter (1990 ▶).
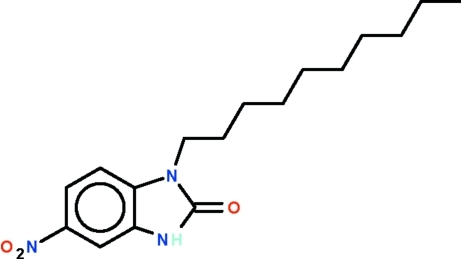


## Experimental

### 

#### Crystal data

C_17_H_25_N_3_O_3_
*M* *_r_* = 319.40
Triclinic, 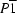

*a* = 5.4933 (2) Å
*b* = 10.3063 (4) Å
*c* = 16.1655 (6) Å
α = 106.504 (2)°
β = 98.545 (2)°
γ = 96.809 (2)°
*V* = 855.21 (6) Å^3^
*Z* = 2
Mo *K*α radiation
μ = 0.09 mm^−1^
*T* = 293 K
0.32 × 0.06 × 0.04 mm

#### Data collection

Bruker APEXII diffractometer
10701 measured reflections
2971 independent reflections
1600 reflections with *I* > 2σ(*I*)
*R*_int_ = 0.068

#### Refinement

*R*[*F* ^2^ > 2σ(*F* ^2^)] = 0.055
*wR*(*F* ^2^) = 0.175
*S* = 0.89
2971 reflections
209 parameters
H-atom parameters constrained
Δρ_max_ = 0.30 e Å^−3^
Δρ_min_ = −0.31 e Å^−3^

### 

Data collection: *APEX2* (Bruker, 2005 ▶); cell refinement: *SAINT* (Bruker, 2005 ▶); data reduction: *SAINT*; program(s) used to solve structure: *SHELXS97* (Sheldrick, 2008 ▶); program(s) used to refine structure: *SHELXL97* (Sheldrick, 2008 ▶); molecular graphics: *X-SEED* (Barbour, 2001 ▶); software used to prepare material for publication: *publCIF* (Westrip, 2010 ▶).

## Supplementary Material

Crystal structure: contains datablocks global, I. DOI: 10.1107/S1600536811004399/zs2093sup1.cif

Structure factors: contains datablocks I. DOI: 10.1107/S1600536811004399/zs2093Isup2.hkl

Additional supplementary materials:  crystallographic information; 3D view; checkCIF report

## Figures and Tables

**Table 1:** Hydrogen-bond geometry (Å, °)

*D*—H⋯*A*	*D*—H	H⋯*A*	*D*⋯*A*	*D*—H⋯*A*
N1—H1⋯O1^i^	0.88	1.92	2.784 (3)	168

Symmetry code: (i) 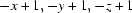.

**Fig. 1.**
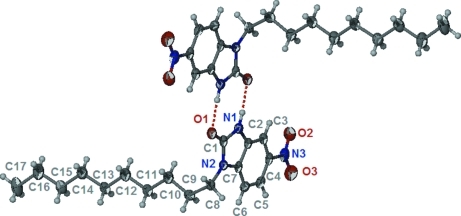
Thermal ellipsoid plot drawn at the 50% probability level (Barbour, 2001), of two molecules of C17H25N3O3 disposed about a center of inversion.

## supplementary crystallographic information

### Comment

Tetraalkylammonium halides are used as phase-transfer catalyst in the synthesis of alkyl-substituted benzimidazolones. A previous study reported the 1-isopropenyl derivative in which the amino –NH unit forms a hydrogen bond to the inversion-related molecule to generate a hydrogen-bonded dimer (Saber *et al.*, 2010). The present compound (Scheme I) features a long *n*-octyl chain that adopts an extended zigzag conformation (Fig. 1). The benzimidazolone part of the C_17_H_25_N_3_O_3_ molecule is planar (r.m.s. deviation 0.016 Å) and its mean plane is aligned at 7.9 (4) ° with respect to the mean plane of the nitro substituent. Two molecules are disposed about a center of inversion to generate a hydrogen-bonded cyclic dimer (Table 1), whose hydrogen-bonding motif is described by the *R*^2^_2_(8) graph set (Etter, 1990).

### Experimental

To 5-nitro-1*H*-benzoimidazol-2(3*H*)-one (0.2 g, 1.1 mmol), potassium carbonate (0.30 g, 2.2 mmol) and tetra-*n*-butylammonium bromide (0.07 g, 0.2 mmol) in DMF (15 ml) was added 1-bromo-*n*-decane (0.46 ml, 2.2 mmol). Stirring was continued at room temperature for 6 h. The salt was removed by filtration and the filtrate concentrated under reduced pressure. The residue was separated by chromatography on a column of silica gel with ethyl acetate/hexane (1/2) as eluent. The compound was recrystallized from diethyl ether to give colorless crystals.

### Refinement

Carbon-bound H-atoms were placed in calculated positions (C—H 0.93–0.97, N–H 0.88 Å) and were included in the refinement in the riding model approximation, with *U*_iso_(H) set to 1.2–1.5*U*_eq_(C,*N*).

### Crystal data

**Table:**

C_17_H_25_N_3_O_3_	*Z* = 2
*M**_r_* = 319.40	*F*(000) = 344
Triclinic, *P*1	*D*_x_ = 1.240 Mg m^−^^3^
Hall symbol: -P 1	Mo *K*α radiation, λ = 0.71073 Å
*a* = 5.4933 (2) Å	Cell parameters from 1521 reflections
*b* = 10.3063 (4) Å	θ = 2.7–27.3°
*c* = 16.1655 (6) Å	µ = 0.09 mm^−^^1^
α = 106.504 (2)°	*T* = 293 K
β = 98.545 (2)°	Prism, colorless
γ = 96.809 (2)°	0.32 × 0.06 × 0.04 mm
*V* = 855.21 (6) Å^3^	

### Data collection

**Table:**

Bruker APEXII diffractometer	1600 reflections with *I* > 2σ(*I*)
Radiation source: fine-focus sealed tube	*R*_int_ = 0.068
graphite	θ_max_ = 25.0°, θ_min_ = 3.9°
φ and ω scans	*h* = −6→6
10701 measured reflections	*k* = −10→12
2971 independent reflections	*l* = −19→19

### Refinement

**Table:**

Refinement on *F*^2^	Secondary atom site location: difference Fourier map
Least-squares matrix: full	Hydrogen site location: inferred from neighbouring sites
*R*[*F*^2^ > 2σ(*F*^2^)] = 0.055	H-atom parameters constrained
*wR*(*F*^2^) = 0.175	*w* = 1/[σ^2^(*F*_o_^2^) + (0.1056*P*)^2^] where *P* = (*F*_o_^2^ + 2*F*_c_^2^)/3
*S* = 0.89	(Δ/σ)_max_ = 0.001
2971 reflections	Δρ_max_ = 0.30 e Å^−^^3^
209 parameters	Δρ_min_ = −0.31 e Å^−^^3^
0 restraints	Extinction correction: *SHELXL97* (Sheldrick, 2008), Fc^*^=kFc[1+0.001xFc^2^λ^3^/sin(2θ)]^-1/4^
Primary atom site location: structure-invariant direct methods	Extinction coefficient: 0.034 (6)

### Fractional atomic coordinates and isotropic or equivalent isotropic displacement parameters (Å^2^)

**Table:**

	*x*	*y*	*z*	*U*_iso_*/*U*_eq_	
O1	0.6868 (3)	0.57512 (19)	0.61197 (12)	0.0413 (6)	
O2	0.8896 (4)	−0.0690 (2)	0.29737 (16)	0.0579 (7)	
O3	1.2510 (4)	−0.0845 (2)	0.36526 (16)	0.0680 (7)	
N1	0.7157 (4)	0.3830 (2)	0.49844 (15)	0.0357 (6)	
H1	0.5863	0.3832	0.4592	0.043*	
N2	0.9999 (4)	0.4426 (2)	0.61946 (15)	0.0337 (6)	
N3	1.0691 (5)	−0.0264 (2)	0.35868 (19)	0.0482 (7)	
C1	0.7879 (5)	0.4765 (3)	0.57918 (19)	0.0354 (7)	
C2	0.8734 (5)	0.2871 (3)	0.48616 (18)	0.0328 (7)	
C3	0.8717 (5)	0.1718 (3)	0.41760 (19)	0.0371 (7)	
H3	0.7483	0.1435	0.3671	0.044*	
C4	1.0672 (5)	0.1002 (3)	0.4288 (2)	0.0380 (7)	
C5	1.2516 (5)	0.1396 (3)	0.5022 (2)	0.0397 (8)	
H5	1.3801	0.0890	0.5052	0.048*	
C6	1.2493 (5)	0.2538 (3)	0.5717 (2)	0.0385 (7)	
H6	1.3723	0.2805	0.6223	0.046*	
C7	1.0566 (5)	0.3268 (3)	0.56298 (19)	0.0347 (7)	
C8	1.1434 (5)	0.5230 (3)	0.70567 (18)	0.0399 (7)	
H8A	1.0786	0.6078	0.7254	0.048*	
H8B	1.3157	0.5469	0.7007	0.048*	
C9	1.1361 (5)	0.4479 (3)	0.77434 (19)	0.0420 (7)	
H9A	1.1954	0.3618	0.7530	0.050*	
H9B	1.2519	0.5027	0.8276	0.050*	
C10	0.8830 (5)	0.4174 (3)	0.7976 (2)	0.0457 (8)	
H10A	0.7678	0.3575	0.7457	0.055*	
H10B	0.8185	0.5023	0.8171	0.055*	
C11	0.8982 (5)	0.3489 (3)	0.8699 (2)	0.0463 (8)	
H11A	0.9593	0.2632	0.8491	0.056*	
H11B	1.0202	0.4076	0.9204	0.056*	
C12	0.6562 (6)	0.3190 (3)	0.8998 (2)	0.0516 (9)	
H12A	0.5896	0.4036	0.9179	0.062*	
H12B	0.5366	0.2556	0.8505	0.062*	
C13	0.6824 (6)	0.2585 (3)	0.9749 (2)	0.0492 (8)	
H13A	0.8060	0.3210	1.0234	0.059*	
H13B	0.7457	0.1731	0.9561	0.059*	
C14	0.4445 (6)	0.2305 (3)	1.0082 (2)	0.0535 (9)	
H14A	0.3754	0.3146	1.0235	0.064*	
H14B	0.3244	0.1634	0.9608	0.064*	
C15	0.4751 (6)	0.1780 (3)	1.0874 (2)	0.0531 (9)	
H15A	0.5400	0.0926	1.0717	0.064*	
H15B	0.5981	0.2439	1.1344	0.064*	
C16	0.2378 (7)	0.1537 (4)	1.1214 (2)	0.0692 (11)	
H16A	0.1189	0.0825	1.0760	0.083*	
H16B	0.1659	0.2371	1.1331	0.083*	
C17	0.2764 (8)	0.1116 (4)	1.2043 (3)	0.0862 (13)	
H17A	0.1189	0.0965	1.2220	0.129*	
H17B	0.3888	0.1832	1.2503	0.129*	
H17C	0.3460	0.0286	1.1932	0.129*	

### Atomic displacement parameters (Å^2^)

**Table:**

	*U*^11^	*U*^22^	*U*^33^	*U*^12^	*U*^13^	*U*^23^
O1	0.0513 (12)	0.0398 (11)	0.0402 (12)	0.0189 (10)	0.0180 (10)	0.0148 (10)
O2	0.0723 (16)	0.0459 (14)	0.0531 (16)	0.0082 (12)	0.0178 (14)	0.0095 (12)
O3	0.0794 (17)	0.0556 (14)	0.0770 (18)	0.0342 (13)	0.0326 (14)	0.0149 (13)
N1	0.0405 (13)	0.0385 (13)	0.0363 (15)	0.0144 (11)	0.0142 (11)	0.0176 (13)
N2	0.0368 (13)	0.0354 (13)	0.0344 (14)	0.0097 (10)	0.0119 (11)	0.0151 (12)
N3	0.0618 (18)	0.0381 (15)	0.056 (2)	0.0155 (14)	0.0303 (16)	0.0194 (14)
C1	0.0446 (17)	0.0345 (16)	0.0357 (18)	0.0080 (14)	0.0186 (14)	0.0179 (15)
C2	0.0383 (16)	0.0332 (16)	0.0355 (18)	0.0072 (13)	0.0170 (14)	0.0184 (14)
C3	0.0440 (17)	0.0374 (16)	0.0355 (18)	0.0054 (13)	0.0146 (14)	0.0172 (15)
C4	0.0491 (18)	0.0321 (16)	0.0444 (19)	0.0096 (14)	0.0273 (16)	0.0192 (15)
C5	0.0412 (17)	0.0383 (17)	0.051 (2)	0.0139 (14)	0.0212 (16)	0.0215 (16)
C6	0.0371 (16)	0.0431 (17)	0.0436 (19)	0.0086 (13)	0.0137 (14)	0.0226 (16)
C7	0.0415 (16)	0.0346 (16)	0.0376 (18)	0.0073 (13)	0.0212 (14)	0.0191 (14)
C8	0.0435 (16)	0.0414 (17)	0.0352 (18)	0.0068 (13)	0.0102 (14)	0.0114 (15)
C9	0.0466 (17)	0.0414 (17)	0.0382 (18)	0.0102 (14)	0.0075 (14)	0.0119 (14)
C10	0.0541 (19)	0.0456 (18)	0.044 (2)	0.0130 (15)	0.0156 (16)	0.0185 (16)
C11	0.0561 (19)	0.0483 (18)	0.0399 (19)	0.0123 (15)	0.0140 (15)	0.0183 (16)
C12	0.064 (2)	0.0493 (19)	0.050 (2)	0.0120 (16)	0.0166 (17)	0.0237 (17)
C13	0.059 (2)	0.0508 (19)	0.043 (2)	0.0115 (16)	0.0125 (16)	0.0199 (16)
C14	0.060 (2)	0.056 (2)	0.054 (2)	0.0110 (16)	0.0165 (17)	0.0275 (18)
C15	0.070 (2)	0.0456 (18)	0.046 (2)	0.0063 (16)	0.0131 (18)	0.0188 (17)
C16	0.086 (3)	0.071 (2)	0.068 (3)	0.017 (2)	0.036 (2)	0.037 (2)
C17	0.136 (4)	0.076 (3)	0.063 (3)	0.012 (3)	0.041 (3)	0.038 (2)

### Geometric parameters (Å, °)

**Table:**

O1—C1	1.240 (3)	C10—C11	1.525 (4)
O2—N3	1.228 (3)	C10—H10A	0.9700
O3—N3	1.231 (3)	C10—H10B	0.9700
N1—C1	1.353 (3)	C11—C12	1.508 (4)
N1—C2	1.382 (3)	C11—H11A	0.9700
N1—H1	0.8800	C11—H11B	0.9700
N2—C1	1.379 (3)	C12—C13	1.511 (4)
N2—C7	1.382 (3)	C12—H12A	0.9700
N2—C8	1.454 (4)	C12—H12B	0.9700
N3—C4	1.468 (4)	C13—C14	1.513 (4)
C2—C3	1.374 (4)	C13—H13A	0.9700
C2—C7	1.402 (4)	C13—H13B	0.9700
C3—C4	1.391 (4)	C14—C15	1.520 (4)
C3—H3	0.9300	C14—H14A	0.9700
C4—C5	1.367 (4)	C14—H14B	0.9700
C5—C6	1.380 (4)	C15—C16	1.509 (4)
C5—H5	0.9300	C15—H15A	0.9700
C6—C7	1.382 (3)	C15—H15B	0.9700
C6—H6	0.9300	C16—C17	1.517 (5)
C8—C9	1.526 (4)	C16—H16A	0.9700
C8—H8A	0.9700	C16—H16B	0.9700
C8—H8B	0.9700	C17—H17A	0.9600
C9—C10	1.514 (4)	C17—H17B	0.9600
C9—H9A	0.9700	C17—H17C	0.9600
C9—H9B	0.9700		
			
C1—N1—C2	110.5 (2)	C9—C10—H10B	109.3
C1—N1—H1	124.7	C11—C10—H10B	109.3
C2—N1—H1	124.7	H10A—C10—H10B	108.0
C1—N2—C7	109.0 (2)	C12—C11—C10	115.4 (2)
C1—N2—C8	124.0 (2)	C12—C11—H11A	108.4
C7—N2—C8	126.9 (2)	C10—C11—H11A	108.4
O2—N3—O3	123.7 (3)	C12—C11—H11B	108.4
O2—N3—C4	118.5 (2)	C10—C11—H11B	108.4
O3—N3—C4	117.8 (3)	H11A—C11—H11B	107.5
O1—C1—N1	127.5 (3)	C13—C12—C11	113.7 (2)
O1—C1—N2	125.5 (3)	C13—C12—H12A	108.8
N1—C1—N2	107.0 (2)	C11—C12—H12A	108.8
C3—C2—N1	132.1 (3)	C13—C12—H12B	108.8
C3—C2—C7	121.8 (2)	C11—C12—H12B	108.8
N1—C2—C7	106.1 (2)	H12A—C12—H12B	107.7
C2—C3—C4	115.5 (3)	C12—C13—C14	115.2 (2)
C2—C3—H3	122.3	C12—C13—H13A	108.5
C4—C3—H3	122.3	C14—C13—H13A	108.5
C5—C4—C3	123.6 (3)	C12—C13—H13B	108.5
C5—C4—N3	118.8 (3)	C14—C13—H13B	108.5
C3—C4—N3	117.6 (3)	H13A—C13—H13B	107.5
C4—C5—C6	120.7 (2)	C15—C14—C13	115.0 (3)
C4—C5—H5	119.6	C15—C14—H14A	108.5
C6—C5—H5	119.6	C13—C14—H14A	108.5
C7—C6—C5	117.2 (3)	C15—C14—H14B	108.5
C7—C6—H6	121.4	C13—C14—H14B	108.5
C5—C6—H6	121.4	H14A—C14—H14B	107.5
N2—C7—C6	131.5 (3)	C16—C15—C14	114.5 (3)
N2—C7—C2	107.3 (2)	C16—C15—H15A	108.6
C6—C7—C2	121.2 (3)	C14—C15—H15A	108.6
N2—C8—C9	113.2 (2)	C16—C15—H15B	108.6
N2—C8—H8A	108.9	C14—C15—H15B	108.6
C9—C8—H8A	108.9	H15A—C15—H15B	107.6
N2—C8—H8B	108.9	C15—C16—C17	113.5 (3)
C9—C8—H8B	108.9	C15—C16—H16A	108.9
H8A—C8—H8B	107.8	C17—C16—H16A	108.9
C10—C9—C8	115.7 (2)	C15—C16—H16B	108.9
C10—C9—H9A	108.4	C17—C16—H16B	108.9
C8—C9—H9A	108.4	H16A—C16—H16B	107.7
C10—C9—H9B	108.4	C16—C17—H17A	109.5
C8—C9—H9B	108.4	C16—C17—H17B	109.5
H9A—C9—H9B	107.4	H17A—C17—H17B	109.5
C9—C10—C11	111.6 (2)	C16—C17—H17C	109.5
C9—C10—H10A	109.3	H17A—C17—H17C	109.5
C11—C10—H10A	109.3	H17B—C17—H17C	109.5
			
C2—N1—C1—O1	−179.0 (2)	C8—N2—C7—C6	2.5 (4)
C2—N1—C1—N2	1.4 (3)	C1—N2—C7—C2	−0.7 (3)
C7—N2—C1—O1	180.0 (2)	C8—N2—C7—C2	−177.4 (2)
C8—N2—C1—O1	−3.2 (4)	C5—C6—C7—N2	179.3 (3)
C7—N2—C1—N1	−0.4 (3)	C5—C6—C7—C2	−0.8 (4)
C8—N2—C1—N1	176.4 (2)	C3—C2—C7—N2	−177.8 (2)
C1—N1—C2—C3	177.4 (3)	N1—C2—C7—N2	1.4 (3)
C1—N1—C2—C7	−1.7 (3)	C3—C2—C7—C6	2.3 (4)
N1—C2—C3—C4	179.3 (2)	N1—C2—C7—C6	−178.4 (2)
C7—C2—C3—C4	−1.7 (4)	C1—N2—C8—C9	114.0 (3)
C2—C3—C4—C5	−0.3 (4)	C7—N2—C8—C9	−69.7 (3)
C2—C3—C4—N3	178.0 (2)	N2—C8—C9—C10	−65.3 (3)
O2—N3—C4—C5	172.5 (2)	C8—C9—C10—C11	−177.1 (2)
O3—N3—C4—C5	−7.0 (4)	C9—C10—C11—C12	177.9 (3)
O2—N3—C4—C3	−5.8 (4)	C10—C11—C12—C13	−176.8 (3)
O3—N3—C4—C3	174.7 (2)	C11—C12—C13—C14	178.5 (3)
C3—C4—C5—C6	1.8 (4)	C12—C13—C14—C15	−176.4 (3)
N3—C4—C5—C6	−176.5 (2)	C13—C14—C15—C16	178.6 (3)
C4—C5—C6—C7	−1.2 (4)	C14—C15—C16—C17	−175.7 (3)
C1—N2—C7—C6	179.2 (3)		

### Hydrogen-bond geometry (Å, °)

**Table:**

*D*—H···*A*	*D*—H	H···*A*	*D*···*A*	*D*—H···*A*
N1—H1···O1^i^	0.88	1.92	2.784 (3)	168

Symmetry codes: (i) −*x*+1, −*y*+1, −*z*+1.
